# Editorial: A Focus on *Actinobacteria*: Diversity, Distribution, and Secondary Metabolites

**DOI:** 10.3389/fmicb.2022.902360

**Published:** 2022-05-05

**Authors:** Yu-Qin Zhang, Xin-Peng Tian, Louis S. Tisa, Imen Nouioui, Wen-Jun Li

**Affiliations:** ^1^Institute of Medicinal Biotechnology, Chinese Academy of Medical Sciences and Peking Union Medical College, Beijing, China; ^2^Key Laboratory of Marine Bio-resources Sustainable Utilization, Guangdong Key Laboratory of Marine Materia Medica, South China Sea Institute of Oceanology, Chinese Academy of Sciences, Guangzhou, China; ^3^Department of Molecular, Cellular, and Biomedical Sciences, University of New Hampshire, Durham, NH, United States; ^4^German Collection of Microorganisms and Cell Cultures GmbH (DSMZ), Braunschweig, Germany; ^5^State Key Laboratory of Biocontrol, Guangdong Provincial Key Laboratory of Plant Resources and Southern Marine Science and Engineering Guangdong Laboratory (Zhuhai), School of Life Sciences, Sun Yat-sen University, Guangzhou, China

**Keywords:** *Actinobacteria*, diversity, biosynthesis gene clusters, secondary metabolites, bioactive compounds

*Actinobacteria* are the most closely related prokaryotic microorganisms of significance to humans. Apart from their significant contribution to soil organic matter turnover (Crowther et al., 2019), they are critical as symbionts in plant-associated microbial communities (Barka et al., 2015), plant-promoting and biocontrol agents (Toumatia et al., 2016; Zahra et al., 2020), and prolific producers of useful natural compounds (Demain and Sanchez, 2009; Idris et al., 2017). As a result, an increasing number of studies have concentrated on the diversity and distribution of actinobacterial resources, as well as the application of novel technologies to explore these microorganisms (Qin et al., 2016). *Actinobacteria* are so intriguing and exciting that has led to considerable novel species, genera, and even higher taxa being proposed and their corresponding novel resources have been collected from diverse ecosystems in recent years (https://lpsn.dsmz.de/phylum/actinobacteria).

After more than a decade of focusing on the diversity of *Actinobacteria* in desert environments, we discovered that members of the family *Geodermatophilaceae* were ubiquitous in various micro-ecosystems in Tengger and Badain Jaran deserts, and that this group of microorganisms played a critical role in determining the bacterial community structure in deserts (Sun et al., 2015, 2018; Jiang et al., 2021). Based on these studies, we hypothesized that *Geodermatophilaceae* represent desert-specific microorganisms and may serve as model organisms for studying the *Actinobacteria*'s resistance to multiple environmental pressures in extreme environments. Thus, we launched the topic “*A Focus on Actinobacteria: Diversity, Distribution, and Secondary Metabolites*” to bring together newly related research findings and ideas to demonstrate the diversity of *Actinobacteria* and their survival mechanisms in various ecosystems, and then to expound on useful *Actinobacteria* in detail.

We are indebted to all the authors for their contributions to this topic, which included one review and eight original research reports. These articles focused on the diversity, function, and biotechnology of *Actinobacteria* from desert, marine, gut, and nodule environments, as well as the application of gene manipulation technologies and metagenome analysis methods to other microorganisms.

Xie and Pathom-aree systematically reviewed recent advances on the technologies to the recovery of novel taxa, ecological functions, and the discovery of previously un-hitherto biotechnological properties of desert *Actinobacteria*. (i) After comparing the efficiency of different actinobacterial isolation techniques, this review strongly recommended that *Actinobacteria* can be isolated from desert associated samples using the sprinkling technique. (ii) Whole-genome sequencing was not only effective at resolving taxonomic bottlenecks, but also provided a wealth of information about the ecology and biotechnology potential of desert species. (iii) The PLate Coverage Algorithm (PLCA), a culture-enriched metagenomic approach, may be used to improve the recovery of microbial diversity on any low-abundance microbial communities. This approach was also beneficial in elucidating the mechanisms by which microbial communities interact with their hosts. (iv) Advances in computational techniques and bioinformatics tools have resulted in an enormously richer understanding of *Actinobacteria* biology in desert environments.

Liu et al. further supported the preceding review. They described an investigation into the diversity, novelty, and pharmacological potential of actinobacterial strains isolated from the Taklamakan desert. A batch of newly isolates, representing novel taxa or exhibiting antagonistic activity against “ESKAPE” pathogens, merit further investigation and exploration. Multi-strategies, including high-throughput screening, small-scale fermentation using deep-plates, One Strain Many Compounds (OSMAC), and compound dereplication *via* UPLC-QToF-MS/MS, were proposed to thoroughly investigate the functional *Actinobacteria*.

Distinct from the desert environments, the deep-sea ecological environment has generaly been regarded as another underexplored habitats for undiscovered, potentially pharmaceutical microbial resources. The orders *Gaiellales* and *Rubrobacterales* were discovered to be widespread in marine ecosystems, despite their difficulty in laboratory cultivation (Chen et al.). To obtain their pure cultures, Chen et al. optimized the growth conditions, and found that light, expanding culture time, and low nutrition could promote *Rubrobacterales* survival on media, and some marine factors are indispensable for their growth.

Guerrero-Garzón et al. investigated the antibiotic activity and comparative genomics of *Streptomyces* spp. isolated from marine sponges. The findings indicated that the dominant actinobacterial genus *Streptomyces* is not only a prolific producer of useful natural compounds, but also an ideal model for Synthetic Biology techniques to mine the selected biosynthesis gene clusters (BGCs).

*Streptomyces* spp. derived compounds have benefited humans for such a long time that it is becoming increasingly difficult to discover new compounds from *Streptomyces* continuously. As a result, it is critical to innovate perspectives, technologies, and strategies for conducting systematic investigations into *Streptomyces*' potential to produce novel compounds. The endophytic *Streptomyces* strain YINM00001, has shown to be strong antimicrobial activity and multiple antibiotic resistances, was predicted as a promising candidate to discover valuable secondary metabolites by genome mining, and proven by using OSMAC approach (Liu et al. ).

Besides above approaches, genetic manipulation was another popular strategy for enhancing *Actinobacteria*'s application potential. The genetic manipulation system for genome modification in the rare actinomycete *Pseudonocardia alni* Shahu was carried out, allowing for the delivery of the powerful CRISPR-Cas machinery into this bacterium *via* this approach (Li et al.). This work developed a toolkit to facilitate the development and improvement of strain *P. alni* Shahu, which served as a useful reference for the development of genetic manipulation methods in other rare actinomycetes.

Culture-independent techniques revealed that *Actinobacteria* dominated in nodule environment of Tunisia, with *Frankia* spp. most frequently being detected (Ghodhbane-Gtari et al.). Along a gradient of aridity ranging from humid to arid, *Frankia*'s absolute and relative prevalence decreased at semi-arid and arid sampling locations. Most abundant secondary metabolite biosynthetic gene clusters were predicted in genomes of *Frankia* sp. Therefore, as the niche builder of root nodules, the functional *Frankia* microsymbiont played a keystone role in the nodule environments, especially in shaping and maintaining the diversity and stability of nodule communities.

The core of research on actinobacterial resources is to discover new compounds to combat the growing spread of drug resistance. Mahilkar et al. developed a quantitative model to examine a population's response to two temporal environmental cues and predicted variables that may be relevant for anticipatory regulatory response evolution. After approximately 850 generations of alternating rhamnose and paraquat environments while checking the experimental evolution of *Escherichia coli*, they concluded that pre-exposure to rhamnose resulted in increased fitness in paraquat environment. This anticipatory regulation is encoded by mutations in global regulators, as revealed by genome sequencing. This study advanced our understanding of how the environment shapes the topology of an organism's regulatory networks.

The study by Mao et al. discovered a significantly altered microbial composition in Parkinson's disease (PD) patients by examining the composition of the gut microbiome in PD patients using shotgun metagenomic sequencing. The Cluster of Orthologous Groups protein database, the KEGG Orthology database, and carbohydrate-active enzymes gene category analysis revealed that branched-chain amino acid–related proteins were significantly increased in the PD group, while GH43 was significantly decreased. Functional analysis of the metagenome confirmed differences in microbiome metabolism between the PD and non-PD groups, specifically in the metabolism of short-chain fatty acid precursors.

We are pleased to present this Research Topic in Frontiers in Microbiology. We sincerely hope that readers will find this Research Topic interesting. Surely, we will constantly contribute to the research on *Actinobacteria* and the journal Frontiers in Microbiology.

## Author Contributions

All authors listed have made a substantial, direct, and intellectual contribution to the work and approved it for publication.

## Funding

This work has been funded by CAMS Innovation Fund for Medical Sciences (CIFMS, 2021-I2M-1-055), National Natural Science Foundation of China (32170021 and 31670010), and Beijing Natural Science Foundation (5212018).

## Conflict of Interest

The authors declare that the research was conducted in the absence of any commercial or financial relationships that could be construed as a potential conflict of interest.

## Publisher's Note

All claims expressed in this article are solely those of the authors and do not necessarily represent those of their affiliated organizations, or those of the publisher, the editors and the reviewers. Any product that may be evaluated in this article, or claim that may be made by its manufacturer, is not guaranteed or endorsed by the publisher.

## References

[B1] Barka E. A. Vatsa P. Sanchez L. Gaveau-Vaillant N. Jacquard C. Meier-Kolthoff J. P.. (2015). Taxonomy, physiology, and natural products of actinobacteria. Microbiol. Mol. Biol. Rev. 80, 1–43. 10.1128/MMBR.00019-15 26609051 PMC4711186

[B2] Crowther T. W. van den Hoogen J. Wan J. Mayes M. A. Keiser A. D. Mo L.. (2019). The global soil community and its influence on biogeochemistry. Science. 365, eaav0550. 10.1126/science.aav0550 31439761

[B3] Demain A. L. Sanchez S. (2009). Microbial drug discovery: 80 years of progress. J. Antibiot. 62, 5–16. 10.1038/ja.2008.16 19132062 PMC7094699

[B4] Idris H. Goodfellow M. Sanderson R. Asenjo J. A. Bull A. T. (2017). Actinobacterial rare biospheres and dark matter revealed in habitats of the chilean atacama desert. Sci. Rep. 7, 8373. 10.1038/s41598-017-08937-4 28827739 PMC5566421

[B5] Jiang Z. M. Zhang B. H. Sun H. M. Zhang T. Yu L. Y. Zhang Y. Q. (2021). Properties of *Modestobacter deserti* sp. nov., a kind of novel phosphate-solubilizing actinobacteria inhabited in the desert biological soil crusts. Front. Microbiol. 12, 742798. 10.3389/fmicb.2021.742798 34803963 PMC8602919

[B6] Qin S. Li W. J. Dastager S. G. Hozzein W. N. (2016). Editorial: *Actinobacteria* in special and extreme habitats: diversity, function roles, and environmental adaptations. Front. Microbiol. 7, 1415. 10.3389/fmicb.2016.01415 27660627 PMC5014857

[B7] Sun H. M. Zhang T. Yu L. Y. Sen K. Zhang Y. Q. (2015). Ubiquity, diversity and physiological characteristics of *Geodermatophilaceae* in shapotou national desert ecological reserve. Front. Microbiol. 6, 1059. 10.3389/fmicb.2015.01059 26483778 PMC4588033

[B8] Sun Y. Shi Y. L. Wang H. Zhang T. Yu L. Y. Sun H.. (2018). Diversity of bacteria and the characteristics of *Actinobacteria* community structure in Badain Jaran Desert and Tengger Desert of China. Front. Microbiol. 9, 1068. 10.3389/fmicb.2018.01068 29875762 PMC5974926

[B9] Toumatia O. Compant S. Yekkour A. Goudjal Y. Sabaou N. Mathieu F.. (2016). Biocontrol and plant growth promoting properties of *Streptomyces mutabilis* strain IA1 isolated from a saharan soil on wheat seedlings and visualization of its niches of colonization. South Afr. J. Bot. 105, 234–239. 10.1016/j.sajb.2016.03.020

[B10] Zahra T. Hamedi J. Mahdigholi K. (2020). Endophytic *Actinobacteria* of a halophytic desert plant *pteropyrum olivieri*: promising growth enhancers of sunflower. Biotech. 3, 10, 514. 10.1007/s13205-020-02507-8 33184598 PMC7649202

